# Beyond Vertigo: Vestibular, Aural, and Perceptual Symptoms in Vestibular Migraine

**DOI:** 10.1007/s11916-024-01245-3

**Published:** 2024-05-23

**Authors:** Claire E. J. Ceriani

**Affiliations:** Department of Neurology, Jefferson Headache Center, Thomas Jefferson University, 900 Walnut St., Ste 200, Philadelphia, PA 19107 USA

**Keywords:** Vestibular migraine, Vertigo, Persistent postural-perceptual dizziness (PPPD), Mal-de-debarquement syndrome (MDDS), Tinnitus, Hearing loss, Otalgia, Alice in Wonderland syndrome (AIWS)

## Abstract

**Purpose:**

To review the vestibular, aural, and perceptual symptoms of vestibular migraine (VM) that may present alongside vertigo.

**Recent Findings:**

Increased research attention to the wide spectrum of symptoms presenting in VM patients has improved understanding of this disorder, with recent identification of five different VM phenotypes. Research into the clinical overlap between VM and other chronic vestibular syndromes such as persistent postural-perceptual dizziness and mal-de-debarquement syndrome reveals a range of vestibular symptoms and hints at pathophysiological connections between migraine and vestibular dysfunction. Studies of migraine treatment for hearing loss suggest patients presenting with aural symptoms may have an underlying diagnosis of migraine and deserve a trial of migraine preventives. Research into the neurologic basis of the perceptual disorder Alice in Wonderland syndrome has revealed brain areas that are likely involved and may help explain its prevalence in VM patients.

**Summary:**

VM is a sensory processing disorder that presents with more than just vertigo. Understanding the range of potential symptoms improves diagnosis and treatment for migraine patients whose diagnosis may be missed when only the symptoms identified in the diagnostic criteria are considered.

## Introduction

**Fig. 1:**
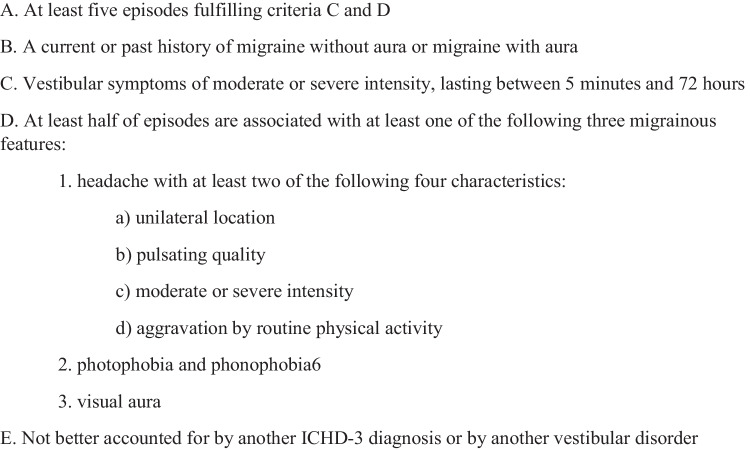
ICHD-3 Diagnostic Criteria for Vestibular Migraine

Vestibular migraine (VM) is the most common cause of episodic vertigo in children and adults [1]. This migraine subtype presents with vestibular symptoms associated with migraine symptoms. It is listed in the appendix of the International Classification of Headache Disorders, 3rd edition (ICHD-3) (Fig. 1) [2]. There are five vestibular symptoms recognized by the ICHD-3 diagnostic criteria: (1) spontaneous internal vertigo (false sense of self-motion) or external vertigo (false sense that the visual surround is spinning or flowing), (2) positional vertigo (occurring after a change in head position), (3) visually induced vertigo (triggered by complex or large moving visual stimuli), (4) head motion-induced vertigo (occurring during head motion), and (5) head motion-induced dizziness with nausea (sensation of disturbed spatial orientation during head motion). In addition to vertigo, many other symptoms are associated with VM, including tinnitus, ear fullness or pain, hearing loss, and Alice in Wonderland syndrome (AIWS) [3••, 4••, 5••, 6••]. Other forms of vestibular dysfunction, such as persistent postural-perceptual dizziness (PPPD) and mal-de-debarquement syndrome (MDDS), may also be seen [6••].

The current diagnostic criteria do not fully capture the spectrum of VM symptoms, and some patients may not meet the criteria and yet have much in common with those who do. A recent study of patients presenting with vertigo and migrainous features found that nearly half of patients did not meet ICHD-3 criteria for VM, but were very similar to the cohort that did meet criteria in nearly every qualitative metric used to describe them [7]. A cluster analysis study of 244 with definite VM as defined by diagnostic criteria identified 5 different clusters or phenotypes of VM: (1) patients with longer duration vertigo attacks, (2) patients with absence of migrainous headache and cochlear symptoms during vertigo, (3) patients with cochlear symptoms during vertigo but not headache, (4) patients with both cochlear and migrainous headache during vertigo, and (5) patients with migrainous headache but no cochlear symptoms during vertigo [5••]. The observation of these distinct subgroups, some with cochlear symptoms as a defining part of their phenotype, suggests that VM patients might be identified more easily when asked about more than just the presence of vertigo during headache. When evaluating patients for a potential diagnosis of VM, it is important to look for symptoms commonly seen in these patients that may be missed if the clinician’s focus is on vertigo alone.

## Vestibular Symptoms

Though the ICHD-3 criteria require attacks of vestibular symptoms lasting between 5 min and 72 h, many patients have persistent vestibular symptoms in between more acute attacks of vertigo. About half of VM patients report almost constant interictal dizziness, with about two-thirds of those experiencing PPPD and a smaller subset experiencing MDDS [6••]. The presence of continuous or near-continuous vestibular symptoms does not negate a diagnosis of VM.

### Persistent Postural-Perceptual Dizziness (PPPD)

PPPD is a chronic vestibular disorder with diagnostic criteria defined in 2017 by the Barany Society (an international society for neuro-otology and vestibular research). This disorder presents with non-spinning vertigo, dizziness, or unsteadiness occurring on most days for at least 3 months, and worsened by movement, upright posture, and exposure to moving or complex visual stimuli [8]. PPPD may be comorbid with migraine, with a recent study of 36 patients reporting that 53% met ICHD-3 criteria for migraine and 17% met criteria for VM specifically [9•]. Nearly a third of the remaining patients met a majority of the migraine criteria. It has also been observed that VM may be a precipitating condition for PPPD in 16% of PPPD patients [10]. It is the author’s experience that some patients with initially episodic VM attacks will go on to develop daily, less severe vestibular symptoms that resemble PPPD, just as patients with episodic migraine who progress to chronic migraine may develop a daily, less severe headache in between acute attacks. PPPD treatment is underresearched, but there is evidence supporting vestibular therapy, cognitive behavioral therapy, selective serotonin reuptake inhibitors (SSRIs), and selective norepinephrine reuptake inhibitors (SNRIs) [11].

### Mal-de-Debarquement Syndrome (MDDS)

MDDS is a vestibular disorder characterized by a prolonged (> 1 month) and inappropriate sensation of movement after exposure to motion, such as sea, air, or train travel. The sensation may be of rocking, swaying, or imbalance, and often improves with re-exposure to motion [12]. MDDS has been recognized since the mid-twentieth century when long-distance travel became accessible to more people, but it is much more recently recognized as occurring spontaneously without a motion trigger as well. A 2018 study of patients with motion-triggered MDDS and non-triggered MDDS found that both groups had a higher population baseline prevalence of migraine (23% and 38%, respectively) [13]. In a 2021 study comparing patients diagnosed with both MDDS and VM with patients diagnosed with MDDS only, MDDS/VM patients reported more interictal visually induced and head motion-induced dizziness, more interictal aural symptoms, and more disability and job resignations [14•]. A small study of 15 patients with MDDS who were treated with a VM management protocol (lifestyle changes, verapamil, nortriptyline, topiramate, or a combination) found that 11 (73%) responded well to migraine treatment [15]. Benzodiazepines, SSRIs, SNRIs, antiepileptic drugs, and migraine preventive vitamins may also be effective [13, 14•].

There is some evidence that MDDS and migraine may share some underlying pathophysiology. In addition to a comorbid association between the disorders and treatment responses to similar medications, neurochemical and hormonal changes may affect these diagnoses in similar ways. MDDS is more common in women, with symptoms often worsening with menses and improving with pregnancy and with many female patients first developing symptoms around menopausal age [16]. It is also theorized that calcitonin gene-related peptide (CGRP) may play a role in MDDS pathophysiology, as CGRP targets the vestibular end organs and is expressed in the vestibular efferent neurons and central vestibular neurons [17].

## Aural Symptoms

The inner ear is connected via neurovascular branches to the trigeminal neurovascular system [18]. Activation of the trigeminal neurovascular system during migraine may in turn cause neurovascular changes in the inner ear and cause aural symptoms. There is evidence that the trigeminovascular system may also influence auditory brainstem function, even interictally. A study of women with VM in their interictal period measured auditory brainstem responses and found increased latencies of the frequency following response and lower loudness discomfort thresholds compared to controls, suggesting that VM involves permanent alterations in subcortical auditory pathways [19]. Even without cochlear symptoms, migraine patients may have abnormalities in electrophysiologic testing suggesting subclinical changes in cochlear function and auditory pathways [20, 21]. The cumulative incidence of cochlear disorders in patients with migraine is significantly higher than in those without migraine [22••]. A diagnosis of “cochlear migraine” has even been proposed to describe patients who experience hearing changes and aural fullness with migraine attacks [23]. Aural symptoms are common in VM and may be as bothersome as the vestibular symptoms. A cluster analysis of VM patients found that 18% of patients had a phenotype of cochlear symptoms during vertigo and 23.4% had a phenotype of both cochlear symptoms and migrainous headache during vertigo [5••].

### Tinnitus

Tinnitus is the perception of sound without an external source and may be described as humming, ringing, buzzing, whooshing, or pounding. Tinnitus is reported by about 25–50% of VM patients [3••, 5••, 6••, 24–26]. It is more common in migraine patients with vestibular symptoms, with a study finding that 43.1% of VM patients reported tinnitus versus only 9.4% of episodic migraine patients without vestibular symptoms [27]. The presence of tinnitus has been associated with younger-onset migraine [28]. Tinnitus may occur as part of a VM attack, but may actually occur more frequently during the interictal period [29•]. The exact pathogenesis remains unclear, but animal studies have shown that somatosensory signals from the spinal trigeminal nucleus cancel out self-produced sounds in the dorsal cochlear nucleus, which may play a role in the spontaneous occurrence of tinnitus [30]. Tinnitus is difficult to treat with a lack of strong evidence for pharmacotherapy, but at least partial improvement has been reported with some migraine preventives, including amitriptyline, nortriptyline, sertraline, and gabapentin [30]. Anecdotally, the author has also had success with duloxetine in a few patients.

### Hearing Loss

During VM attacks, some patients describe muffled hearing or hearing loss [3••, 5••, 6••]. A study of VM patients found that 21.1% had hearing loss, which was usually mild and easily reversible low-frequency hearing loss and more frequently associated with tinnitus and aural fullness [25]. Another study found that 92% of VM patients had some degree of extended high-frequency hearing loss and 68% had standard frequency hearing loss, despite only 43% endorsing hearing loss complaints [31•]. A large study comparing over 15,000 migraine patients with controls found an odds ratio in migraine versus controls of 1.74 (*p* < 0.001) for sensorineural hearing loss [32]. A study using the National Health and Nutrition Examination Survey database found that people with migraine were more likely to have subjective hearing loss (25.0% vs 16.6%) and tinnitus (34.6% vs 16.9%) than people without migraine [33•]. This corresponded to migraine having an odds ratio of 1.5 (95% CI 1.3–1.7, *p* < 0.001) for hearing loss and 2.2 (95% CI 2.0–2.4, *p* < 0.001) for tinnitus. The risk for hearing loss in migraine likely increases over time. A study that followed up VM patients after a median of 9 years found that cochlear symptoms had increased from 15% initially to 49% at follow-up, and 18% had developed mild bilateral sensorineural hearing loss involving the low-frequency range [29•].

Sudden sensorineural hearing loss (SSNHL), though rare, has been found to be more prevalent in people with migraine, and migraine preventive drugs may be efficacious in these patients [30]. A study of 10,280 migraine patients and 41,120 matched controls followed over a median of 5 years found that the migraine cohort had a greater risk of developing SSNHL, and comorbidity with hypertension was associated with a trend of developing SSNHL [34]. The pathophysiologic connection between migraine and hearing loss is not known, but it has been suggested that trigeminal activation with release of CGRP and substance P causes vascular dilation in the inner ear, leading to aseptic inflammation that affects inner ear function [35]. Patients with SSNHL who are treated with nortriptyline and topiramate in addition to oral and intratympanic steroids have greater improvements in hearing thresholds than patients treated with steroids alone [36•]. A combination of intratympanic steroid injections with nortriptyline, topiramate, and/or verapamil has also been shown to be beneficial [37•]. Any patient presenting with SSNHL should be offered a trial of migraine preventive therapy.

### Aural Discomfort

Otalgia or aural pressure/fullness is very common, occurring in nearly half of the VM patients [3••, 5••, 6••, 25]. In a case series of 11 patients presenting with at least 6 months of persistent aural fullness, 6 fulfilled ICHD-3 criteria for migraine, and a majority reported visual motion sensitivity, head motion sensitivity, phonophobia, and photophobia [38]. After instructing patients to improve sleep hygiene, follow a “migraine diet,” and begin a migraine preventive (nortriptyline or verapamil), eight patients had complete or near-complete resolution of aural fullness [38]. Of note, this study excluded patients who met diagnostic criteria for VM, but demonstrates that patients presenting with unexplained aural fullness should be screened for migraine in general. A retrospective study of 77 patients presenting with aural fullness and migraine features found that 14% fulfilled diagnostic criteria for migraine, and 74% met a majority of criteria, but there were minimal differences in the overall prevalence of migraine features between these two groups [39•]. This suggests that the diagnostic criteria may be too stringent, and migraine should be considered a possible diagnosis in patients presenting with unexplained aural fullness, even if they do not satisfy all criteria.

Both pediatric and adult patients with migraine are more likely to experience otalgia than patients with other types of headache, and patients with otalgia are more likely to experience headache than patients without otalgia [40, 41]. In a study of patients presenting with unexplained otalgia, 65% met diagnostic criteria for migraine, and 92% improved with first-line acute and preventive migraine therapies (nortriptyline, amitriptyline, topiramate, diltiazem, magnesium, and/or propranolol) [42]. As in aural fullness, clinicians should look for migrainous features in the history of patients presenting with unexplained otalgia and consider treatment with a migraine preventive, even if diagnostic criteria are not fully met.

## Perceptual Symptoms (Alice in Wonderland Syndrome)

Alice in Wonderland syndrome (AIWS) is a perceptual disorder characterized by distortions of visual perception, body schema, and the experience of time [43•]. It has been associated with a broad range of neurologic disorders, psychiatric disorders, infections, medications, and substances, but the most common cause in adults is migraine [43•]. In a study that screened over 200 patients presenting to a headache clinic for AIWS, 19% reported lifetime occurrence (90% of whom had migraine with aura) [44]. All patients reported visual symptoms, with some also reporting somatosensory symptoms. In a study of VM patients, 14% had symptoms of AIWS, with most (47%) reporting visual distortions [45•]. These distortions included illusory splitting (objects appear vertically cleaved down the middle), teleopsia (objects appear far away), underwater vision, xanthopsia (yellow tint washing into the visual field), dolly zoom effect (as would be produced by zooming the camera lens while simultaneously moving the camera farther away), frosted glass vision, enhanced stereoscopic vision (seeing in extreme detail), and closed eye hallucinations. There were also an unusually high proportion of extrapersonal misperceptions (out-of-body experiences, derealization, depersonalization). Some patients also reported somesthetic distortions, such as aschematia (inadequate representation of the space occupied by part of the body) and total body or partial macrosomatognosia or microsomatognosia (body or body parts feeling too big or too small). One patient described time distortion (everything seemed to slow down), and one described auditory distortion (unable to hear her own voice) [45•].

The underlying pathophysiology of AIWS remains unclear. A recent lesion mapping study identified cases of AIWS following brain lesion and found that patients presenting with visual symptoms shared an occipital site of brain damage that was more often right-sided [46]. Patients who had somesthetic symptoms or a mix of symptoms had lesions located in the right hemisphere, including the thalamus, insula, frontal lobe, and hippocampal/parahippocampal cortex. In a fMRI study of migraine patients with AIWS, migraine patients with visual aura, and healthy controls, researchers found a pattern of altered thalamic connectivity in all patients with migraine, but with more profound and diffuse alterations observed in AIWS [47]. In AIWS, there was an increased connectivity between a lateral occipital region and the posterior part of the superior temporal sulcus, which is an area of multisensory integration, including vestibular, auditory, visual, and tactile inputs. A similar study found that migraine with aura and AIWS patients had lower functional connectivity in lateral and medial visual networks, but AIWS patients also had higher connectivity in basal ganglia and executive control networks which positively correlated with migraine frequency [48].

Treatment of AIWS depends on its underlying cause. In patients with migraine, usual migraine preventives, such as antiepileptics, antidepressants, and beta-blockers, may be effective [44]. In VM specifically, topiramate, lamotrigine, clonazepam, nortriptyline, amitriptyline, and venlafaxine have been reported to be helpful [45•].

## Conclusions

Vestibular migraine is an underdiagnosed disorder. It may present with a wide range of symptoms, but only a few are represented in the diagnostic criteria. Familiarity with the other non-vertiginous symptoms may help clinicians recognize this diagnosis more often, especially when vertigo is not the patient’s main concern. As many of these symptoms may present in migraine more generally as well, knowledge of the broad range of possible presentations should improve patient care. Many patients who could benefit from migraine therapy will be missed if only the most common migraine symptoms are assessed.

## Data Availability

No datasets were generated or analyzed during the current study.
